# Developing the National Usability-Focused Health Information System Scale for Physicians: Validation Study

**DOI:** 10.2196/12875

**Published:** 2019-05-16

**Authors:** Hannele Hyppönen, Johanna Kaipio, Tarja Heponiemi, Tinja Lääveri, Anna-Mari Aalto, Jukka Vänskä, Marko Elovainio

**Affiliations:** 1 National Institute for Health and Welfare Helsinki Finland; 2 Aalto University Espoo Finland; 3 Helsinki University Hospital Helsinki Finland; 4 University of Helsinki Helsinki Finland; 5 Finnish Medical Association Helsinki Finland

**Keywords:** physicians, health information systems, questionnaire, validation studies

## Abstract

**Background:**

Problems in the usability of health information systems (HISs) are well acknowledged, but research still lacks a validated questionnaire for measuring and monitoring different dimensions of usability of HISs. Such questionnaires are needed not only for research but also for developing usability of HISs from the viewpoint of end-user experiences.

**Objective:**

This study aimed to develop and test the validity of the questionnaire measuring the National Usability-Focused HIS-Scale (NuHISS) among a nationally representative sample of Finnish physicians.

**Methods:**

We utilized 2 cross-sectional data collected from a random sample of Finnish physicians in 2014 (N=3781; of which 2340 [61.9%] were women) and 2017 (N=4018; of which 2604 [64.8%] were women). Exploratory and confirmatory factor analyses (structural equation modeling [SEM]) were applied to test the structural validity of the NuHISS. As the concurrent validity measure, we used the self-reported overall quality of the electronic health record system (*school grade*) provided by the participants using marginal structural models.

**Results:**

The exploratory factor analyses with Varimax rotation suggested that the 7-factor solution did offer a good fit to the data in both samples (C^2^=2136.14 in 2014 and C^2^=2109.83 in 2017, both *P*<.001). Moreover, structural equation modelling analyses, using comparative fit index (CFI), Tucker-Lewis Index (TLI), Normed Fit Index (NFI), root mean squared error of approximation (RMSEA), and Standardized Root Mean square Residual (SRMR), showed that the 7-factor solution provided an acceptable fit in both samples (CFI=0.92/0.91, TLI=0.92/0.91, NFI=0.92/0.91, RMSEA=0.048/0.049, and SRMR=0.040/0.039). In addition, concurrent validity of this solution was shown to be acceptable. *Ease of use*, but also all other dimensions, was especially associated with overall quality reports independent of measured confounders. The 7-factor solution included dimensions of *technical quality*, *information quality*, *feedback*, *ease of use*, *benefits*, *internal collaboration*, and *cross-organizational collaboration*.

**Conclusions:**

NuHISS provides a useful tool for measuring usability of HISs among physicians and offers a valid measure for monitoring the long-term development of HISs on a large scale. The relative importance of items needs to be assessed against national electronic health policy goals and complemented with items that have remained outside the NuHISS from the questionnaire when appropriate.

## Introduction

Problems in usability of health information systems (HISs) are well acknowledged in research [1]. The vast investments in the adoption of HISs in the United States as well as in Europe have been driven by expectations reflecting key usability goals, particularly increased effectiveness and efficiency in health care [2-4]. The clinician community has, while considering electronic health record (EHR) systems as an improvement over the paper-based system, expressed frustration with the level of usability of available systems as well as their support for information exchange [1,5]. Moreover, several studies suggest that HISs may cause stress and frustration to clinicians and this appears to have increased recently [6,7]. If clinicians cannot achieve their goals with efficiency, effectiveness, and satisfaction by using the implemented information systems (ISs), they seek alternative solutions to reach their goals, that is, by using paper to document and transfer health information [8]. Declining to use is one important indication that the anticipated benefits are not being realized.

Research has shown that with high-quality management and high perceived usability, HISs could yield significant quality and productivity gains [4]. To show evidence of these gains arising from HISs use, appropriate measures need to be used. However, usability is qualitative and multidimensional by nature, thereby challenging the measuring [9] and thereby accumulation of results [10] on a larger scale. Depending on the definition used, the attributes and metrics of measurement have varied [11-14]. The widely known definition for usability is defined by the International Organization for Standardization (ISO) standard as follows: “usability refers to an extent to which a system, product or service can be used by specified users to achieve specific goals with effectiveness, efficiency and satisfaction in a specified context of use” [13]. The possible measures for each of these 3 aspects of usability are many. On the basis of a review of practices in measuring usability by Hornbæk [9], the challenges include, for example, distinguishing and empirically comparing subjective and objective measures of usability, studying long-term use and usability, extending measures of satisfaction beyond postuse questionnaire, validating and standardizing the host of subjective satisfaction questionnaires used, and studying correlations between usability measures as a means for validation. Alongside with usability, user experience (UX) as a concept has gained interest and is described as a person’s perceptions and responses resulting from the use or anticipated use of a product, system, or service [13]. According to the ISO standard [13], usability criteria can be used to assess aspects of UX as well.

Although governments have pushed the adoption of electronic Health (eHealth) systems and services, they have often lacked knowledge of longer term and larger scale eHealth usability and UX in health care contexts. Both the Organisation for Economic Co-operation and Development (OECD) and European Union (EU) have included information and communication technology (ICT) benchmarking as one of the issues on their policy agendas [15,16]; however, current HIS monitoring on a national level focuses on availability and usage rate of key functionalities of EHRs, personal health record (PHR) systems, health information exchange (HIE), and telemedicine [17-19]. Some HIS-related health care output or efficiency indicators (eg, number of visits saved, impact on length of stay, time saved by system use, and cost saved by system use) have also been defined [20] but rarely used in systematic, large-scale eHealth monitoring.

Validated usability questionnaires (eg, Software Usability Measurement Inventory (SUMI) [21], System Usability Scale (SUS) [22], and Questionnaire for User Interaction Satisfaction (QUIS) [23]) are context and domain independent and focus on evaluating the usability of user interfaces typically after usability testing. Our aim was to study and monitor longer-term experiences on usability and experienced outcomes of complex HISs from the viewpoint of physicians and their clinical tasks in specific contexts of clinical work. In addition, we aimed at addressing the development of the EHR systems. SUMI, SUS, or QUIS questions were therefore not as such regarded suitable as repeated measures of perceived usability of HISs on a national level.

The large-scale eHealth monitoring tools developed by the EU and OECD [17,24] include indicators for availability of key HIS functionalities and information contents, for example, documentation and retrieval of patient data, availability of patient summary data, and medication list. They do not cover end-users’ experiences on usability or experienced benefits of these functionalities or information contents.

The DeLone and McLean ISs success model that was developed in 1992 and revised in 2003 [25,26] is based on vast theoretical and empirical IS research with validation and updates. It has been used in over 30 scientific publications and, more importantly, also applied on a national level in the health care context in the Canada Health Infoway evaluation framework for HISs [20,27-29]. The original model and its application in the eHealth evaluation framework in Canada offer 6 interrelated dimensions on IS success: (1) System quality, (2) Information quality, (3) Service quality, (4) Use, (5) User satisfaction, and (6) Benefits. The framework does not list specific items or measures. The subareas are not specific to key functionalities and information contents needed in clinical work.

The Finnish eHealth strategy from 2015 listed a national-level eHealth usability survey as one strategic means to reach the strategy objectives [30]. In 2012, Nordic eHealth research network was established under the Nordic Council of Ministers to develop common Nordic eHealth indicators. The Finnish HIS usability-focused questionnaire for physicians was adopted in Iceland and Denmark in 2018. The OECD is also following work by the Nordic countries on common eHealth indicators for countries with advanced national ICT infrastructures [30].

The need to collect national-level evidence on usability and UXs of HISs in health care contexts to steer national eHealth policies and implementation coupled with the lack of suitable measures led to development of the Finnish *EHR systems as tools for physicians* questionnaire and within it, the National Usability-Focused HIS Scale (NuHISS) for physicians in 2009. The development team consisted of 2 physician-researchers, 1 usability researcher, and 2 researchers with experience in sociology of technology and national benchmarking studies. The first questionnaire for physicians with NuHISS was conducted in 2010, before implementation of the first national eHealth service—ePrescription. The second data collection round in 2014 was timed so that the national ePrescription service was in full use in the public sector and implementation of the national patient data repository (Kanta) was to begin. In 2017, the data collection was repeated for physicians for the third time and also extended to cover the nurses. The data collection was timed so that the Kanta system was in full use in Finland. The Ministry of Social Affairs and Health has agreed to fund the next data collection round in 2020, extending it further to social workers and asking for a plan beyond 2020.

Establishing the Finnish questionnaire containing the NuHISS as a regular source of evidence for steering national eHealth policies as well as increased international attention about the Finnish questionnaire has added pressure for validation of the key measures used for usability. Thus, the objective of this study was to develop a valid and reliable measure of NuHISS for physicians. The research questions were as follows:

1. What are the most important dimensions of usability and UXs of HISs that the NuHISS for physicians should measure?
2. Does the developed NuHISS show acceptable construct validity (do the selected survey items measure their corresponding dimension and can the dimensions be separated from each other) and concurrent validity (do the dimensions have associations to other factors, such as quality ratings, that they theoretically should) of the scale?

## Methods

### The Survey and Data Used to Validate the Usability-Focused HIS Scale

#### The Survey Population

This report is based on the data collected from a random sample of Finnish physicians at 2 data collection points (2 cross-sectional samples) in 2014 (N=18,257) and 2017 (N=18,326). The response rates were 21% in 2014 and 22% in 2017 providing analytic samples of 3781 in 2014 and 4018 in 2017 [5,31]. Owing to missing values in a part of the variables, we imputed both datasets (missing pattern of both the samples is reported in the Multimedia Appendix 1). We used the multiple imputation method with chained equations using Mice R-package. We generated 5 separate imputed datasets for analyses purposes. The study has been approved by the Finnish National Institute for Health and Welfare Ethics Board.

#### The Survey Questionnaire

Key EHR, HIE, and PHR functionalities and information needed by physicians in everyday clinical work (eg, documentation, viewing and retrieval of patient data, medication list, decision support, HIE, and patient-provided data) were used as a starting point of the questionnaire; see the OECD model survey *The OECD Guide for Measuring ICTs in the Health Sector* [17,18]). The questions were grounded in physicians’ key HIS use tasks to increase face validity of the questions and to increase comparability with HIS availability monitoring (including the OECD model survey). The usability-focused questions (including aspects of stability, reaction speed, ease-of-use, recovery from errors, learnability, availability, information quality, and utility of information as well as effects) were modified based on the Finnish Medical Association’s information technology (IT)-physicians’ listing of key strengths and weaknesses of their HIS, as well as a comprehensive analysis of existing usability constructs [eg, 11-14,20-23,25,26]. The full questionnaire included 17 background questions, 41 usability-focused items, a list of 17 most urgent EHR development needs to select from, a list of 15 best functioning EHR features to select from, and a 9-item module measuring HIS-related well-being. For manager-level physicians, there was an additional 11-item module measuring HIS support for management.

#### The Usability-Focused HIS-Scale Items Used

Of the 41 usability-focused items, 32 items that were identical in the 2014 and 2017 questionnaires were selected for the NuHISS to measure physicians’ experiences on usability and benefits of HISs. The scale items are depicted in Table 1.

#### Items Used for Concurrent Validity Evaluation

As the concurrent validity measure of the dimensions of our construct, we selected the self-reported overall quality of the EHR system (*school grade*) provided by the participants. The overall quality was rated by a continuous scale from 4=*very poor* to 10=*excellent*. The scale was dichotomized into low (7 or less) and high (more than 7) quality estimates. In 2014, 1095 of 3781 respondents (29%) and in 2017 altogether 1323 of 4018 respondents (33%) rated the system they used as a high-quality system.

In the concurrent validity analyses, gender, the year of birth, work tenure (in years), and whether the respondent was specialized (yes/no) were used as time independent covariates and overall system quality (measured by school grade given to the system) as the outcome variable (Table 2).

**Table 1:** Measured items in the National Usability-Focused HIS-Scale scale.

Short name	Questionnaire item (with 5-point Likert scale: 1=fully disagree, 5=fully agree)
Logic	The arrangement of fields and functions is logical on computer screen
Terminology	Terminology on the screen is clear and understandable (eg, titles and labels)
Documenting	Entering and documenting patient data is quick, easy, and smooth
Operating info	The systems keep me clearly informed about what it is doing (eg, saving data)
Straightforward tasks	Routine tasks can be performed in a straight forward manner without the need for extra steps using the system
Needed patient data	It is easy to obtain necessary patient information using the EHR^a^ system
Nursing record	The information on the nursing record is in easily readable format
Stability	The systems are stable in terms of technical functionality (does not crash and no downtime)
System errors	Faulty system function has caused or has nearly caused a serious adverse event for the patient
Reaction speed	The system responds quickly to inputs
Unexpected actions	In my view, the system frequently behaves in unexpected or strange ways
Missing info	Information entered/documented occasionally disappears from the IS^b^
Medic list quality	The patient’s current medication list is presented in a clear format
Summary view	The EHR system generates a summary view (eg, on a timeline) that helps to develop an overall picture of the patients’ health status
Order completion	The system monitors and notifies when the orders given to nurses have been completed
Patient-provided info	Measurement results provided electronically by the patient (eg, via patient portal) help to improve the quality of care
Collaboration	EHR systems support cooperation and communication between physicians and patients
Suggestion implementation	The system supplier implements suggested corrections and amendments as wished
Vendor interest	The system supplier is interested in feedback from users
Implementation speed	Suggestions for corrections and amendments are implemented sufficiently quickly
Care quality	ISs help to improve quality of care
Care continuity	ISs help to ensure continuity of care
Guideline adherence	ISs support compliance and adherence with the treatment recommendations
Medication errors	ISs help in preventing errors and mistakes associated with medications
Duplicate tests	ISs help to avoid duplicate tests and examinations
Care needs and impacts	The EHR system provides me with information about the need for and effectiveness of treatment of my patients
HIE^c^ medication	Information on medications ordered in other organizations is easily available
HIE speed	Obtaining patient information from another organization often takes too much time
HIE data quality	Patient data (also from other organizations) are comprehensive, up-to-date, and reliable
HIE collaboration	EHR systems support cooperation and communication between physicians working in different organizations
Professionals collaboration	EHR systems support cooperation and communication between physicians and nurses
Physician collaboration	EHR systems support cooperation and communication between physicians in your own organization

^a^EHR: electronic health record.

^b^IS: information system.

^c^HIE: health information exchange.

**Table 2:** Demographics, including means and SDs of overall system quality (school grade) covariates in the 2014 and 2017 data.

Variable		Year of data collection	
		2014	2017
Target population, n		16,350	17,210
Questionnaire sent (target population), %		91	93
Respondents		3781	4018
**Gender**			
	Male, %	38.1	35.1
	Female, %	61.9	64.8
	Mean (SD)	1.62 (0.49)	1.65 (0.48)
**Age (years), %**			
	<34	16.9	16.7
	35-44	21.2	21.9
	45-54	28.4	26.7
	55>	33.6	34.7
Year of birth, mean		1966	1969
**Specialization**			
	Not specialized (1), %	33.1	32.6
	Specialized (2), %	66.9	67.4
	Mean (SD)	1.33 (0.47)	1.33 (0.47)
**Work tenure (experience, years)**			
	<10, %	25.7	25.1
	>10<20, %	22.0	22.1
	>20<30, %	27.1	27.0
	30+, %	25.2	25.7
	Mean (SD)	19.63 (11.42)	19.71 (11.48)
**School grade given to the primary** **electronic health record** **system**			
	10 (excellent)	0.5	0.6
	9	5.2	6.3
	8	20.9	23.5
	7	29.6	32.7
	6	24.2	21.9
	5	14.7	11.2
	4 (fail)	4.9	3.8
	Mean (SD)	6.64 (1.27)	6.82 (1.23)

#### Statistical Analyses Assessing Sample Differences

Means and SDs of the NuHISS items for the 2 time points (2014 and 2017) were calculated and Welch 2-sample *t* test was used to analyze the differences in the mean profile of the responses from the 2 time points. Following the research questions and established psychometric testing procedures we tested the validity of the NuHISS in 2 steps: (1) structural validity test (do the intended dimensions or latent variables explain the covariance of the corresponding items) showing whether the scale measures the defined dimensions and (2) concurrent validity test (are the dimensions associated with the factors they should be associated with).

#### Assessing Construct Validity of the NuHISS Scale Dimensions

The preliminary structural analyses were conducted by calculating bivariate correlations between the 32 study variables in the 2014 and then in the 2017 data. For grouping the NuHISS scale items into dimensions, we then tested the factor structure and number of dimensions using exploratory factor analyses with eigenvalue 1 and loading structure as a criterion for the appropriate number of factors. The factorial validity of the original scales was tested separately among the 2 samples (time points 2014 and 2017) with exploratory factor analyses (Varimax rotation; [32]) and then structural equation modeling (SEM; confirmatory factor analyses).

Second, we tested the structure using SEM [33] that offers more stringent testing and allowing items to be loaded only to their corresponding latent variables (factors). SEM is a multivariate statistical analysis technique. This technique is a combination of factor analysis and multiple regression analysis, and it is used to analyze the structural relationship between measured variables and latent constructs. Goodness-of-fit of the SEM models was evaluated based on the chi-square test (X^2^), RMSEA, CFI, TLI, and Akaike’s information criterion (AIC). A nonsignificant chi-square value indicates that the model is a good fit to the data. RMSEA values of less than .05 and .08 suggest a good and reasonable fit, respectively. For CFI and TLI, values above .90 and .95 represent an acceptable and a good fit, respectively. AIC is a measure used to compare any models that have the same set of variables. In such cases, the model with the smaller value of AIC will be preferred [34]. Testing the final structure was done in 3 steps. First, a 1-factor model was estimated where all remaining items were loaded on the same underlying dimension (null model). In the second step, a model representing the original theoretical model was estimated, and, in the final step, the structural invariance (test showing whether the structure can be considered as similar between 2 measurement points) was tested between 2 samples as strong/scalar invariance with factor loadings and intercepts constrained to be similar. Again the same fit indexes were used as in the overall SEM test.

As a final step for structural validation, we assessed internal consistency of each dimension using Cronbach alpha reliability coefficient. The closer Cronbach alpha coefficient is to 1.0, the greater the internal consistency of the items in the dimension.

#### Assessing Concurrent Validity of the Scale Dimensions

Concurrent validity is the extent to which one measurement is backed up by a related measurement obtained at about the same point in time. In testing, the validity of results obtained from one test (in this case *ease of use*) can often be assessed by comparison with a separate but related measurement (in this case, school grade given to the system) collected at the same point in time [35]. Concurrent validity was tested separately for each dimension of the EHR system with the overall quality (measured by school grade given to the system) evaluation as the criteria. The associations between overall quality evaluation and each dimension were tested using the marginal structural model (MSM) approach proposed by Robins et al [36,37], with inverse probability weights taking into account the effects of potential covariates. The approach produces a pseudopopulation with balance in all included covariates.

Statistical programming language R (version 3.5.1)/R-studio and multiple statistical R-packages (psych, psycho, missForest, mice, miceadds, ggplot2, resahpe2, lavaan, semPlot, semTools, ipw, sandwich, and survey) were used for the statistical analyses.

## Results

### Differences of Samples From the 2 Time Points

**Figure 1:**
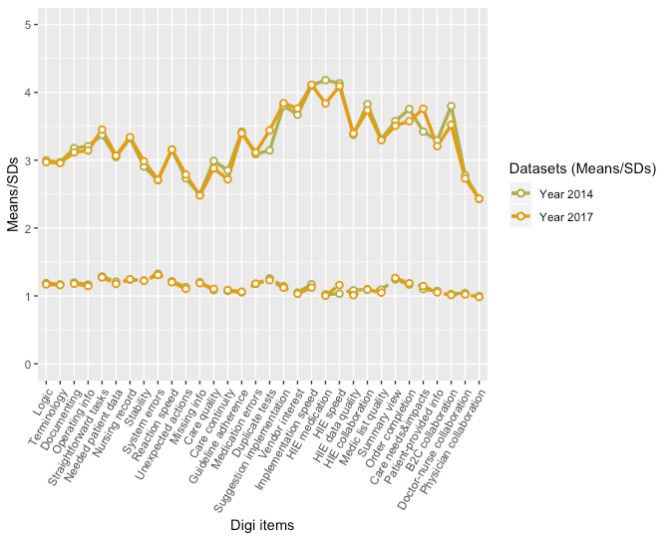
Means and standard deviations of the measured items in 2014 and 2017. HIE: health information exchange.

Means and SDs of the measured items in 2014 and 2017 are presented in Figure 1. There were some differences between time points especially in HIE medication (*information on medication prescribed in other organizations is easily accessible*), duplicate tests (*ISs help prevent overlapping examinations*), care needs and impacts (*IS provides me information on the need and efficiency of care*), and B2C collaboration (*ISs support collaboration between physicians and patients*). The mean profiles were, however, relatively similar: Overall correlations between samples were 0.97, suggesting that the means were very similar between time points. The Welch 2-Sample *t* test suggested that the difference of the overall means between samples (in 2014, mean 3.28; in 2017, mean 3.27; difference=0.016) was not significant (*t*_(7757.06)_=1.14; 95% CI [–0.012 to 0.044]; *P*>.10).

### Construct Validity of the NuHISS Scale

#### Item-to-Item Correlations

**Figure 2:**
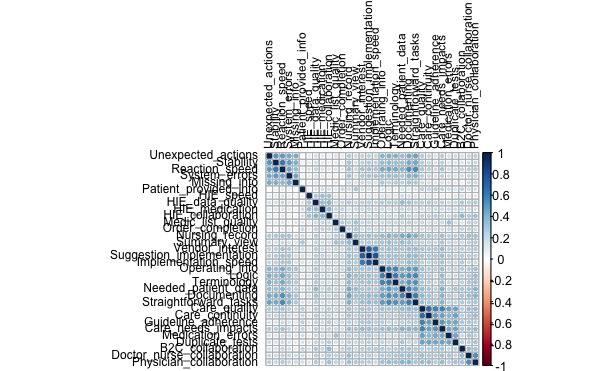
Correlation matrixes among items in 2014.

**Figure 3:**
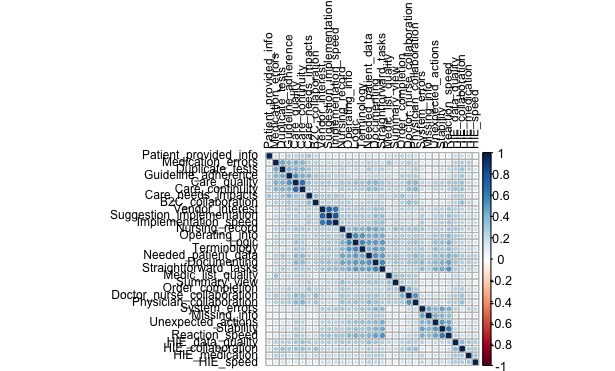
Correlation matrixes among items in 2017.

Correlation matrixes in both samples are presented in Figures 2 and 3. In 2014 data, items around dimension that we named *technical quality* show strong mutual correlations in both years, also correlating especially with most of the relatively strongly mutually correlating items in dimension that we named *ease of use*. *Ease of use* items also correlate with the dimension named *benefit*. Items in the dimension that we named *cross-organization collaboration* also cluster together, but with less significant correlations (lighter color) and correlations also with *internal collaboration*, *ease of use*, and *benefits* items. Items in the *feedback* dimension show strongest between-item correlation, represented by the darkest color, without strong external correlations. Items in the *info quality* dimension do not cluster together so clearly. *Benefit* dimension items correlate clearly with each other. The 2 *internal collaboration* items correlate with each other and also with some of *ease of use* and *cross-organization collaboration* items.

In the 2017 data, the clearest item-to-item correlations are within the *feedback* dimension items. In addition, *ease of use* items, *technical quality* items, *internal collaboration* items, and most of *benefits* dimension items show clear item-to-item correlations. *Cross-organizational collaboration* items show clearer within-item correlations in 2017 than in 2014 data.

#### Scale Structure and Reliability Testing

**Figure 4:**
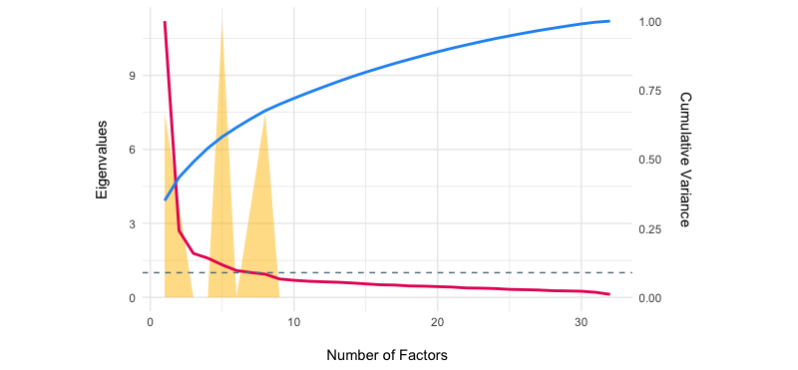
Eigenvalues and explained variance by the number of factors (exploratory factor analyses) in 2014 sample.

**Figure 5:**
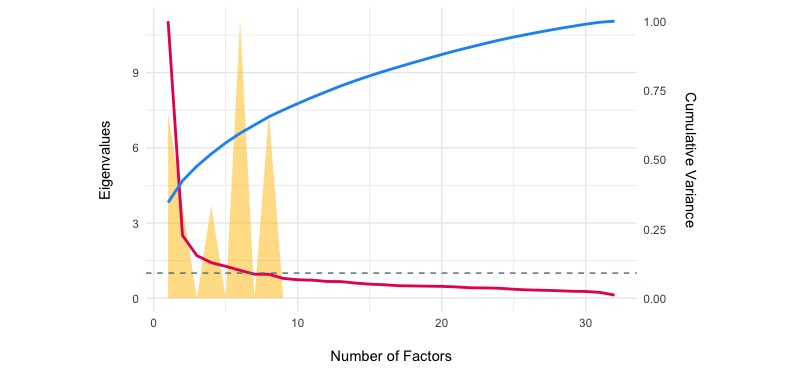
Eigenvalues and explained variance by the number of factors (exploratory factor analyses) in 2017 sample.

The exploratory factor analyses with Varimax rotation suggested that the 7-factor solution did offer a good fit to the data in both samples (test of the hypothesis that 7 factors are sufficient, X^2^_293_=2136.14 in 2014 and X^2^_293_=2109.83 in 2017, both *P*<.001, see also Figures 4 and 5). However, in both samples the choice of 6 factor was supported by 2 (out of 9; 22.22%) methods (Optimal Coordinates and Parallel Analysis) and the choice of 8 factor was supported by 2 (out of 9; 22.22%) methods (Bayesian information criterion [BIC] and Sample Size Adjusted BIC).

**Figure 6:**
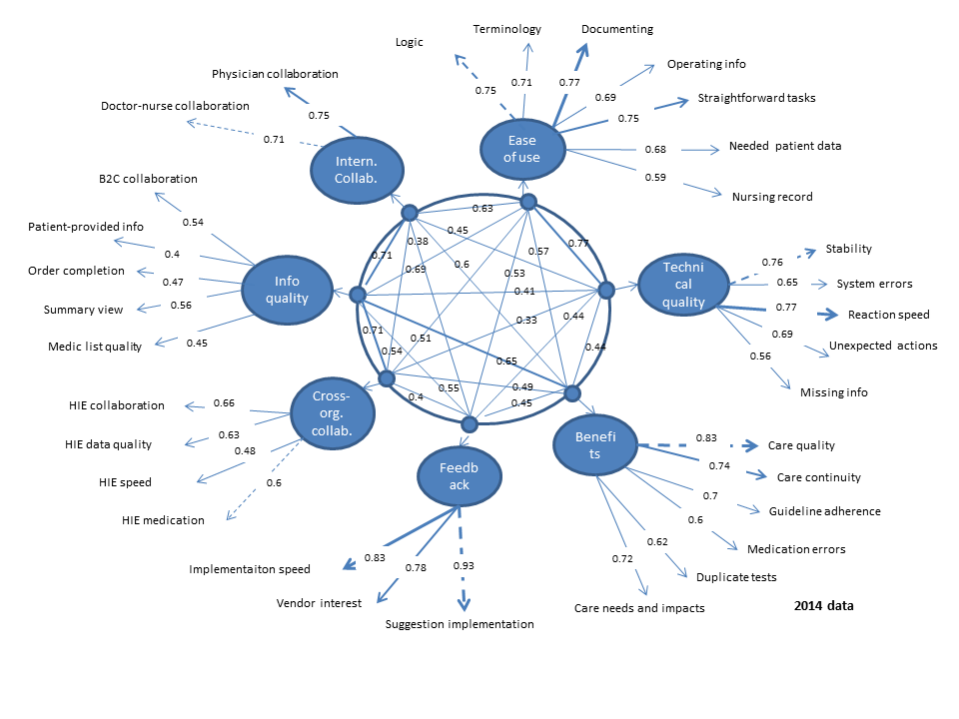
Confirmatory factor analysis in 2014 data.

**Figure 7:**
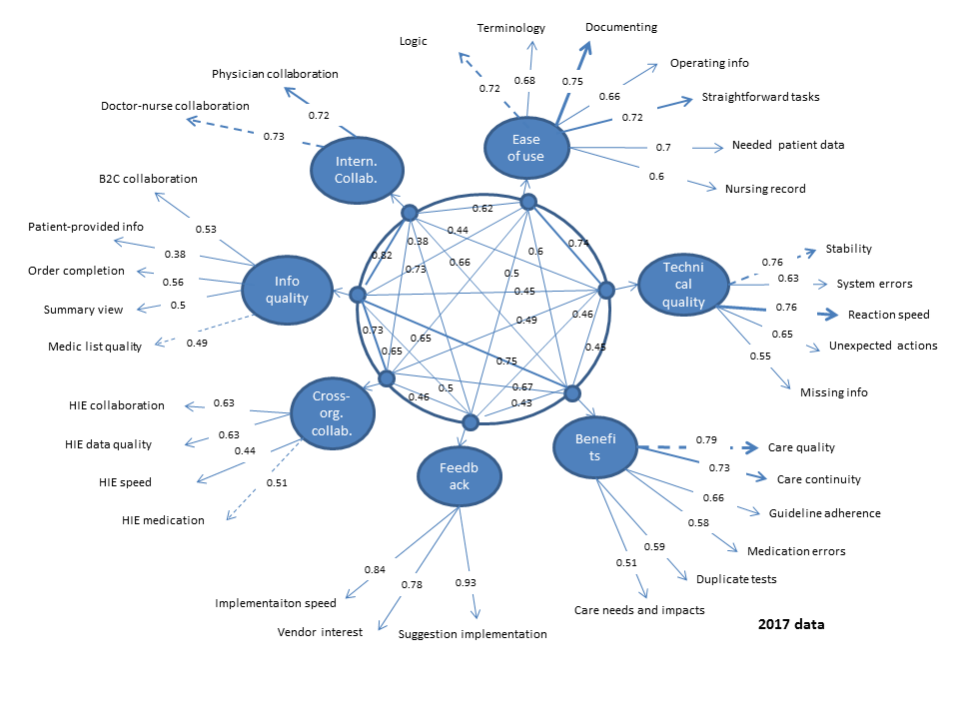
Confirmatory factor analysis in 2017 data.

In SEM analyses, the null model (without predicted structure) did not provide an acceptable fit to the data in 2014 (CFI=0.64, TLI=0.62, NFI=0.64, RMSEA=0.100, and SRMR=0.081) or in 2017 (CFI=0.66, TLI=0.64, NFI=0.66, RMSEA=0.094, and SRMR=0.074). The original 7-factor solution, however, provided an acceptable fit in both samples (CFI=0.92/0.91, TLI=0.92/0.91, NFI=0.92/0.91, RMSEA=0.048/0.049, and SRMR=0.040/0.039). The final solutions are presented in Figures 6 and 7.

The strongest 2 factors (measured by the loadings of items) were *feedback* and *internal collaboration*—in both years all the item loadings were over 0.7. All items in *ease of use*, *technical quality*, and *benefits*—factors had factor loadings over 0.5 both years. The weakest factor loadings in 2017 were in *information quality* factor (0.38 in 2014 for Patient-provided information item, 0.45/0.49 for Medication list quality item, and 0.47/0.56 for Order completion item in 2014/2017). In addition, *cross-organizational collaboration* factor loading for 1 item remained under 0.5 in 2017 (0.44 for HIE speed item). In addition, the internal reliability of the final factors (assessed with alpha coefficients) in the 2014/2017 data shows that the first 4 factors were the strongest:

1. Factor 1 *ease of use* (alpha=.87/.86).
2. Factor 2 *technical quality* (alpha=.82/.80).
3. Factor 3 *benefits* (alpha=.85/.81).
4. Factor 4 *feedback* (alpha=.88/.88).
5. Factor 5 *cross-organizational collaboration* (alpha=.69/.64).
6. Factor 6 *information quality* (alpha=.61/.62).
7. Factor 7 *internal collaboration* (alpha=.70/.69).

As the test of structural consistency, we performed the measurement invariance test that assesses the psychometric equivalence of a construct across groups or across time [38]. Results suggest that the final measure provided only minimal invariance between samples. The sequential tests are reported in Table 3. This was expected, because the fit of the final 7-factor solution was relatively modest.

**Table 3:** Changes of the fit indexes according to the sequential invariance tests.

Invariance tests steps	*Df*	Akaike’s information criterion	Bayesian information criterion	Chi-square	Chi-square difference	*Df* difference	*P*-difference (>chi-square)
fit.configural	886	660,523	662,145	8613.6	—^a^	—	—
fit.loadings	911	660,626	662,075	8766.9	153.29	25	<2.2e-16^b^
fit.intercepts	936	661,458	662,733	9648.7	881.78	25	<2.2e-16^b^
fit.residuals	968	661,693	662,747	9947.7	299.00	32	<2.2e-16^b^
fit.means	975	661,848	662,853	10,116.5	168.86	7	<2.2e-16^b^

^a^Not applicable.

^b^*P*<.001.

### Concurrent Validity of the Scale Items

In the weighted sample, the covariates had a balanced distribution between those who evaluated the overall quality of the system (*school grade*) low and high in both measurement years (Tables 4 and 5). The distribution suggests that the MSM fits relatively well and is effective in balancing covariates across *Overall system quality* (school grade) evaluation sequences (Table 6).

**Table 4:** Distribution of covariates between those who evaluated the overall quality of the electronic health record system (*school grade*) low and high in both measurement years from inverse probability weighting in 2014.

Variable	0 (school grade low; n=3780.96^a^), mean (SD)	1 (school grade high; n=3781.33^a^), mean (SD)	Standardized mean difference
Gender	1.62 (0.49)	1.62 (0.49)	0.007
Year of birth	1966.24 (10.93)	1966.22 (10.84)	0.002
Tenure	19.85 (11.46)	19.86 (11.33)	0.001
Specialized	1.33 (0.47)	1.33 (0.47)	0.003

^a^n refers to pseudopopulation samples from inverse probability weighting.

**Table 5:** Distribution of covariates between those who evaluated the overall quality of the electronic health record system (*school grade*) low and high in both measurement years from inverse probability weighting in 2017.

Variable	0 (school grade low); n=4018.24^a^)	1 (school grade high; n=4017.07)	Standardized mean difference
Gender, mean (SD)	1.65 (0.48)	1.65 (0.48)	0.001
Year of birth, mean (SD)	1969.24 (11.16)	1969.21 (10.83)	0.003
Tenure, mean (SD)	19.74 (11.60)	19.77 (11.26)	0.003
Specialized, mean (SD)	1.33 (0.47)	1.32 (0.47)	0.002

^a^n refers to pseudopopulation samples from inverse probability weighting.

**Table 6:** Controlled direct effect of overall quality evaluation (*school grade*) estimated from the marginal structural model.

Factor		Odds ratio	95% CI
**2014**			
	Ease-of-use	0.29	0.28 to 0.31
	Technical quality	0.36	0.34 to 0.38
	Benefits	0.49	0.46 to 0.52
	Feedback	0.44	0.42 to 0.48
	Cross-organizational collaboration	0.59	0.56 to 0.62
	Information quality	0.58	0.55 to 0.60
	Internal collaboration	0.49	0.46 to 0.52
**2017**			
	Ease-of-use	0.34	0.32 to 0.36
	Technical quality	0.39	0.37 to 0.41
	Benefits	0.55	0.52 to 0.58
	Feedback	0.48	0.45 to 0.51
	Cross-organizational collaboration	0.60	0.57 to 0.63
	Information quality	0.58	0.55 to 0.60
	Internal collaboration	0.54	0.51 to 0.57

### The Validated NuHISS Scale

The study resulted in a validated scale including 7 dimensions: the *technical quality* dimension measures reliability and safety aspects of the EHR system, including 5 items. The 5 items in the *information quality* dimension reflect availability and format of key information types of the EHR system. Information quality measures for the HIE functionality are included in the *cross-organizational collaboration* dimension. The *feedback* dimension measures responsiveness of the EHR system vendor to improvement suggestions including 3 items. The *ease of use* dimension consists of 7 items related to the key functionalities of the EHR system (including reading, documenting, and patient data retrieval). The *benefit* dimension covers 6 items measuring UX on overall benefits of HISs. The *internal collaboration* dimension with its 2 measures is actually a specific benefit that measures how well the EHR system supports cooperation and communication between professionals within their own organization. *Cross-organizational collaboration* dimension is another specific benefit dimension with 4 measures on systems support cooperation and communication among professionals in different organizations (focusing on HIE functionalities). Loadings of each of the measures show the strongest and weakest measures within each dimension (Table 7).

**Table 7:** The validated National Usability-Focused HIS-Scale with dimension reliability and item loadings.

Dimension (reliability 2014/2017) and short name		Item on the questionnaire (with 5-point Likert scale: 1=fully disagree, 5=fully agree)	Factor loading 2014/2017
**Technical quality (alpha=.82/.80)**			
	Stability	The systems are stable in terms of technical functionality (does not crash, no downtime)	0.76/0.76
	System errors	Faulty system function has caused or has nearly caused a serious adverse event for the patient	0.65/0.63
	Reaction speed	The system responds quickly to inputs	0.77/0.76
	Unexpected actions	In my view, the system frequently behaves in unexpected or strange ways	0.69/0.65
	Missing info	Information entered/documented occasionally disappears from the IS	0.56/0.55
**Information quality (alpha=.61/.62)**			
	Medic list quality	The patient’s current medication list is presented in a clear format	0.45/0.49
	Summary view	The EHR system generates a summary view (eg, on a timeline) that helps to develop an overall picture of the patient’s health status	0.56/0.5
	Order completion	The system monitors and notifies when the orders given to nurses have been completed	0.47/0.56
	Patient-provided info	Measurement results provided electronically by the patient (eg, via patient portal) help to improve the quality of care	0.40/0.38
	B2C collaboration	EHR systems support co-operation and communication between physicians and patients	0.54/0.53
**Feedback (alpha=.88/.88)**			
	Suggestion implementation	The system supplier implements suggested corrections and amendments as wished	0.93/0.93
	Vendor interest	The system supplier is interested in feedback from users	0.78/0.78
	Implementation speed	Suggestions for corrections and amendments are implemented sufficiently quickly	0.83/0.84
**Ease of use (alpha=.87/.86)**			
	Logic	The arrangement of fields and functions is logical on computer screen	0.75/0.72
	Terminology	Terminology on the screen is clear and understandable (eg, titles and labels)	0.71/0.68
	Documenting	Entering and documenting patient data is quick, easy and smooth	0.77/0.75
	Operating info	The systems keep me clearly informed about what it is doing (eg, saving data)	0.69/0.66
	Straightforward tasks	Routine tasks can be performed in a straight forward manner without the need for extra steps using the system	0.75/0.72
	Needed patient data	It is easy to obtain necessary patient information using the EHR system	0.68/0.7
	Nursing record	The information on the nursing record is in easily readable format	0.59/0.6
**Benefits (alpha=.85/.81)**			
	Care quality	ISs help to improve quality of care	0.83/0.79
	Care continuity	ISs help to ensure continuity of care	0.74/0.73
	Guideline adherence	ISs support compliance and adherence with the treatment recommendations	0.7/0.66
	Medication errors	ISs help in preventing errors and mistakes associated with medications	0.6/0.58
	Duplicate tests	ISs help to avoid duplicate tests and examinations	0.62/0.59
	Care needs and impacts	The EHR system provides me with information about the need for and effectiveness of treatment of my patients	0.72/0.51
**Cross-organizational collaboration (alpha=** **.69/.64)**			
	HIE medication	Information on medications ordered in other organizations is easily available	0.6/0.51
	HIE speed	Obtaining patient information from another organization often takes too much time	0.48/0.44
	HIE data quality	Patient data (also from other organizations) are comprehensive, up-to-date and reliable	0.63/0.63
	HIE collaboration	EHR systems support co-operation and communication between physicians working in different organizations	0.66/0.63
**Internal collaboration (alpha=.70/.69)**			
	Professionals collaboration	EHR systems support co-operation and communication Between physicians and nurses	0.71/0.73
	Physician collaboration	EHR systems support co-operation and communication Between physicians in your own organization	0.75/0.72

## Discussion

The main aim of this study was to develop and test the validity of the questionnaire measuring NuHISS among a nationally representative sample of Finnish physicians. The exploratory factor analyses showed that the 7-factor solution did offer a good fit to the data and SEM analyses showed that it provided an acceptable fit. Moreover, concurrent validity of this solution was shown to be acceptable. The 7-factor solution included *technical quality*, *information quality*, *feedback*, *ease of use*, *benefits*, *internal collaboration*, and *cross-organizational collaboration* dimensions. Our results show that NuHISS provides a useful tool for measuring the usability of HISs among physicians and offers a valid measure for monitoring the long-term development of HISs on a large scale.

### Differences of Samples

Overall correlations between the 2 samples suggested that the means were very similar between time points. The Welch 2-Sample *t* test verified that the difference was not significant. This generated a good basis for validation of the scale.

### Validity and Reliability of the Scale

Construct validity—the degree to which a test measures what it claims to be measuring—was supported by correlation analysis, which revealed the items clustering together, although correlations for some clusters were stronger and for some others weaker and more dispersed. The underlying components were analyzed using factor analysis—principal components analysis (PCA) for data collected with the same scale in 2 time points. PCA led to a 32-item 7-component solution with 65% of the total variance explained in 2014 and 63% in 2017. In confirmatory factor analyses, the original 7-factor solution provided an acceptable, although modest, fit in both samples. Component or factor loadings revealed that the highest loadings were for all items in the *feedback* factor and many items in the *technical quality*, *ease of use*, *benefits*, and *internal collaboration* factors. The *information quality* factor had overall the lowest loadings.

Internal consistency of factors was assessed by reliability (Cronbach alpha). Reliability of all factors exceeded .60, with the highest alphas in *feedback* (over .80), *ease of use,* and *benefit* factors. The *information quality* factor was the weakest in both years (.61/.62). *Cross-organizational collaboration* reliability was also below .70 in both years.

Concurrent validity measures the extent to which results correspond to those of a previously established measurement of the construct. The covariates had a balanced distribution between those who evaluated the overall quality of the system (school grade) low and high in both measurement years, suggesting that the MSM fits relatively well and is effective in balancing covariates across quality evaluation sequences.

### Comparison of NuHISS and IS Success Model

The final 7-factor solution was compared with IS success model dimensions and items (Canada Infoway 2012 version, Multimedia Appendix 2). NuHISS covered all but one of the IS success model dimensions (Use) and most of the key subareas. The IS success model dimension of *Use* was out of the scope of our survey (Availability and Usage rate are assessed in Finland with a separate survey to health care providers). In addition, the items were grouped in a slightly different manner. Multimedia Appendix 2 compares the 2 scales, where black font shows the actual items for each factor or dimension and gray font location of the item, if different in the scales.

The comparison shows that overall, the NuHISS-scale items are more grounded to the physician work and more detailed than the IS success model items. The NuHISS scale has a more focused *technical quality* factor than the IS success model *System Quality* dimension. In addition to similar items, the latter includes items that fell into NuHISS *ease of use* and *benefits* factors. NuHISS has a dedicated item for *ease of use*, whereas in the IS success model, corresponding items are listed under *system quality* and *information quality* dimensions. NuHISS *benefits* factor and *collaboration* factors correspond to IS success model *user satisfaction* and *information quality* dimensions. NuHISS has specific factors for *cross-organizational* and *internal collaboration*, unlike the IS success model. NuHISS does not include a Service quality factor, although the full questionnaire contains these items.

On the basis of this comparison, *service quality* factor is a potential new factor to be added in the scale in future. In addition, *information quality* factor has the clearest improvement needs based on the weakest reliability and factor loadings.

### Strengths and Limitations

The face validity of our questionnaire is good: experts (physicians using the different HISs in their everyday work) have participated in generating the questionnaire and questions were grounded to the core IT functionalities and information contents needed by physicians. The physicians have evaluated the importance of questions from the viewpoint of actual end-users and long-term use of the systems in clinical environments. The surveys have also been pilot tested on both occasions (before sending out the 2014 and 2017 surveys) on a subset of the intended population. The scale offers a valid tool for measuring usability of HISs among professionals and adds substantially to previous scales such as those focusing on measuring usability, acceptability, and user satisfaction of mobile apps among clients [39-41].

However, the questionnaire may have shortcomings typical to questionnaire-based surveys: it may include questions that are understood differently by different respondents, depending on the experience they have had with different systems and other individual factors. Although trying to use the language familiar to practicing physicians, we have also been obliged to use terminology from user interface and interaction design fields, such as *label*, *input*, or *screen*. In addition, we used the term *EHR* in instances where questions were particularly focused on the respondents’ main EHR system, and *ISs* in instances where questions focused not only on EHRs but also related specialty-specific regional or national *ISs* or patient-provided data (either via the EHR or via standalone systems). The wording aimed at relieving respondents from knowing the particulars of system integration, but may have also confused some respondents. As the clinical ICT system environments often include several systems, which are used simultaneously, it is not always clear for the physicians which functionalities and features are related to EHR systems and which to other IT systems.

The total number of respondents in both surveys was about 4000. Response rates remained relatively low (21% to 22%). A small proportion of this is due to the sampling method: We targeted the survey to all physicians in clinical work. The Finnish Medical association’s membership register did not allow us to select only physicians in clinical work into the target population. Therefore, the questionnaires were sent to all working aged physicians (ie, to a larger target population, with a cover letter calling for responses from physicians in clinical work). Calculation of the response rate was carried out from the sent questionnaires, which included physicians not in clinical work. Comparison of the respondents with the target population in 2014 [5] and 2017 [31] showed good representativeness of samples in both years.

The questionnaire length is another possible reason for the relatively low response rate. Although the core *NuHISS* for physicians had only 32 items, the full questionnaire had 17 background questions, 41 usability-focused items, a list of 17 most urgent EHR development needs to select from, a list of 15 best functioning EHR features to select from, and a 9-item module measuring HIS-related well-being. For manager-level physicians, there was an additional 11-item module measuring HIS support for management.

All the questionnaire items related to IS success were not included in the validated scale. Our questions on system integration and proficiency of use were not included because of a different scale. In the exploratory factor analysis (Multimedia Appendix 3), our items measuring learnability (*Learning to use the EHR system does not require a lot of training*), recovery from errors (*It is easy to correct mistakes [such as entry errors, ending up in the wrong screen, changing incorrect selections, etc]*), and decision support quality (*The reminders, alerts and warnings provided by the system are useful and are adequate*) loaded on the *ease of use* factor but did not qualify there in the validation. In the exploratory factor analysis, our items *Diagnostic imaging results are easily available on a regional level* and *Laboratory results are easily available and are logically presented on a regional level* loaded on *cross-organizational collaboration* factor but did not qualify for the validated scale. In the exploratory factor analysis, our questions *If I have problems with the system I can easily get help* and *Use of EHR systems frequently takes my attention away from the patient* loaded on *technical quality* factor but did not qualify into the validated scale. In the exploratory factor analysis, our item *I know to whom and how I can send feedback on the system, if I so wish* loaded on the *feedback* factor but did not qualify into the validated scale. All these questions reflect aspects of usability that may still be important to keep in the full questionnaire.

Although the functionalities and information needed by doctors in daily patient care remain relatively constant, development of technologies may enhance some functionalities (ie, artificial intelligence-assisted decision support and improved personalization possibilities). These developments should also be considered in the full questionnaire for physicians in future. In addition, further development of some of the measures in the *information quality* factor may be called for. In the exploratory factor analysis, there was an eighth factor, which we named *Business to Client collaboration*. This did not qualify as an individual factor in the validation, but combining the items with the *information quality* factor provided a satisfactory result. The eighth factor may become valid, if some items are added or rephrased.

### Conclusions

To our knowledge, Finland is the first country to have administered regular national monitoring of usability of HISs from the viewpoint of end-user experiences. The introduced tool—NuHISS for physicians—offers a valid measure for monitoring the long-term development of HISs on a large scale. The scale is highly adoptable in other countries—it has already been used in Iceland, Denmark, and Germany in 2018. The relative importance of items needs to be assessed against national eHealth policy goals and complemented with items that have remained outside the NuHISS from the questionnaire when appropriate. Development of HIS functionalities calls for further development of the scale, especially within the *information quality* domain. Similar national-level scales have been developed in Finland for nurses and social workers, based on the physician scale. First data collections were conducted from 2017 to 2018. Validation of these in due course will show generalizability of the physician scale across professional groups.

## Appendix

Multimedia Appendix 1 Number of missing values in each item and missing patterns.

Multimedia Appendix 2 Comparison of National Usability-Focused HIS-Scale and Information System Success model dimensions and items.

Multimedia Appendix 3 Exploratory factor analysis factor matrix with factor loadings.

## Abbreviations

AIC: Akaike’s information criterion
BIC: Bayesian information criterion
CFI: comparative fit index
EHR: electronic health record
eHealth: electronic health
EU: European Union
HIE: health information exchange
HIS: health information system
ICT: information and communication technology
IS: information system
ISO: International Organization for Standardization
IT: information technology
MSM: marginal structural model
NFI: Normed Fit Index
NuHISS: National Usability-Focused HIS-Scale
OECD: Organisation for Economic Co-operation and Development
PCA: principal components analysis
PHR: personal health record
QUIS: Questionnaire for User Interaction Satisfaction
RMSEA: root mean squared error of approximation
SEM: structural equation modeling
SRMR: Standardized Root Mean square Residual
SUMI: Software Usability Measurement Inventory
SUS: System Usability Scale
TLI: Tucker-Lewis Index
UX: user experience
