# Supplementary figures and images for: Developing the National Usability-Focused Health Information System Scale for Physicians: Validation Study
Source: J Med Internet Res. 2019 May 16;21(5):e12875. doi: 10.2196/12875 (PMC6542250; doi:10.2196/12875)

Multimedia Appendix 1: Number of missing values in each item and missing patterns

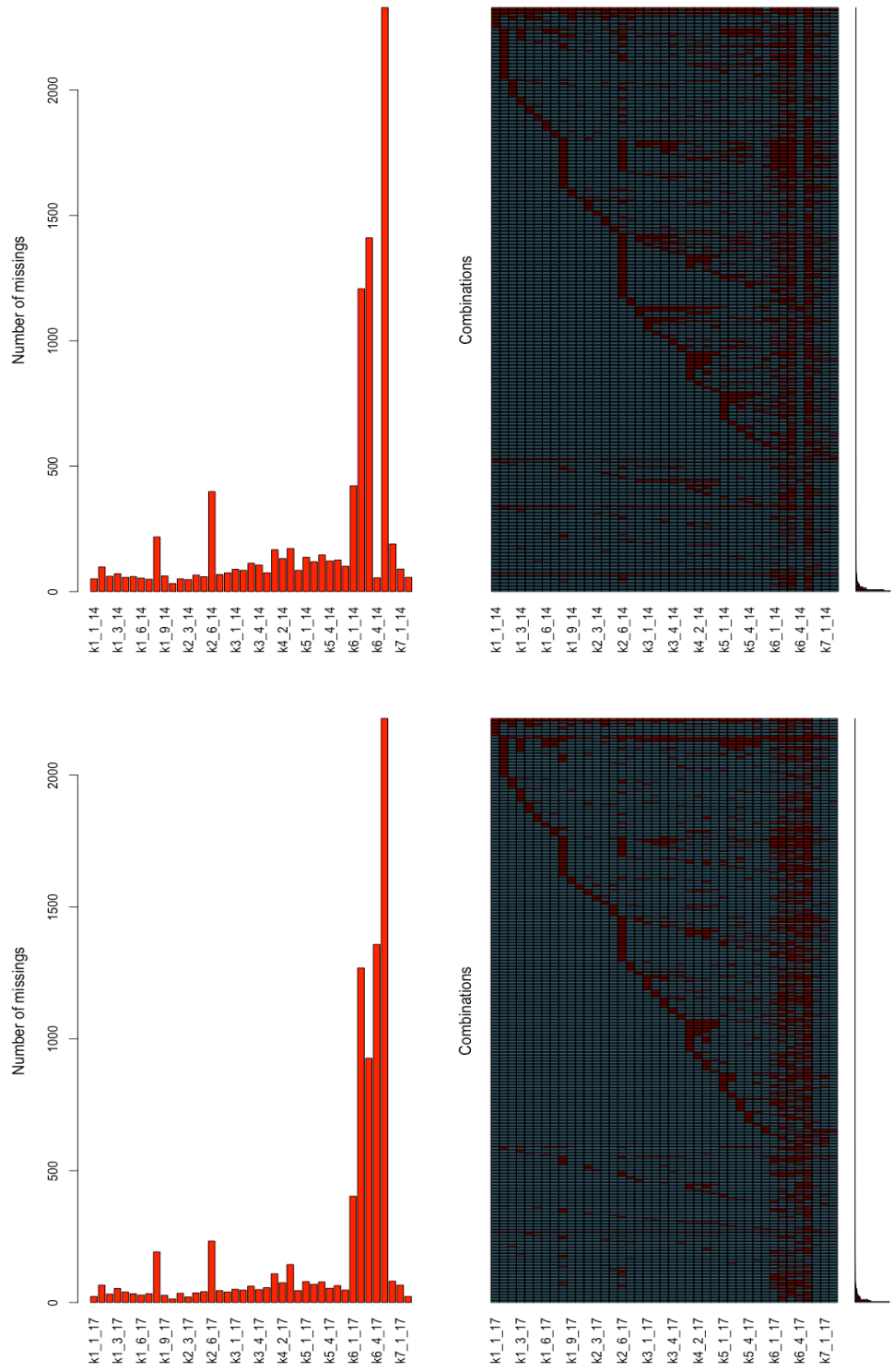

Supplement: Multimedia Appendix 1 [file jmir_v21i5e12875_app1.pdf]
