# Supplementary material for: Developing the National Usability-Focused Health Information System Scale for Physicians: Validation Study
Source: J Med Internet Res. 2019 May 16;21(5):e12875. doi: 10.2196/12875 (PMC6542250; doi:10.2196/12875)
Supplement: Multimedia Appendix 2 [file jmir_v21i5e12875_app2.pdf]

Multimedia Appendix 2: Comparison of NuHISS and IS Success model dimensions and items

NuHISS factors and items	IS success model dimensions and sub-dimensions
Technical quality	System quality
(School grade given to system)	Overall quality of the system
Response time	Response time
Reliability	Reliability
Security	Security
(Ease of use = Separate factor)	Ease of use
(Benefit-factor-items)	Impact on work performance
(Ease of use-factor-items)	Integration into workflow
Ease of Use	(One sub-dimension in System quality-dimension)
Logic	(Information quality-dimension: format and layout of information)
Terminology	(Information quality-dimensionr: format and layout of information)
Documenting	(System quality-dimension: integration into workflow
Operating info	
Straightforward tasks	(System quality-dimension: integration into workflow
Needed patient data	(Information quality-dimension: content relevance)
Nursing record	(Information quality-dimension: format and layout of information)
Benefits	User satisfaction
(School grade given to system)	Overall satisfaction
Duplicate tests	Productivity
(Cross-org. Collaboration: HIE speed)	Productivity
Care quality	Quality of care
Care continuity	Continuity of care
Guideline adherence	Impact on Job
Guideline adherence	Decision making
(Cross-org. Collaboration-factor)	Sharing of patient information
(Internal collaboration-factor)	Sharing of patient information
(Included in full questionnaire)	Efficiency of orders
Medication errors	
Care needs and impacts	
Information quality	Information quality
(Cross-org. Collaboration-factor)	Content completeness and accuracy
(Ease of use-factor: Needed patient data-item)	Content relevance
Medication list quality	Format and layout of information
(Ease of use-factor: Logic)	Format and layout of information
(Ease of use-factor: Terminology)	Format and layout of information
(Ease of use-factor: Nursing record)	Format and layout of information
Summary view	Content relevance

Order completion	(System quality: Integration into workflow
Patient-provided info	Content relevance
B2C collaboration	(System quality: Integration into workflow
(Technical quality-factor: response time)	Speed at which info is provided
(Benefit-factor: HIE speed)	Speed at which info is provided
(Cross-org. Collaboration-factor: HIE medication)	Availability when needed
Cross-organisational collaboration	
HIE medication	(Information quality: Content completeness and accuracy)
HIE speed	(Information quality: Speed at which info is provided)
HIE data quality	(Information quality: Content completeness and accuracy)
HIE collaboration	(User satisfaction: sharing of patient information)
Internal collaboration	
Professional collaboration	(User satisfaction: sharing of patient information)
Physician-physician collaboration	(User satisfaction: sharing of patient information)
	Service quality
In the full questionnaire, not in NuHISS	implementation process
In the full questionnaire, not in NuHISS	Level of training
In the full questionnaire, not in NuHISS	Level of ongoing support
Feedback	
Vendor responsiveness	
