# Supplementary material for: Developing the National Usability-Focused Health Information System Scale for Physicians: Validation Study
Source: J Med Internet Res. 2019 May 16;21(5):e12875. doi: 10.2196/12875 (PMC6542250; doi:10.2196/12875)
Supplement: Multimedia Appendix 3 [file jmir_v21i5e12875_app3.pdf]

Multimedia Appendix 3. Exploratory factor analysis factor matrix with factor loadings

	F1	F2	F3	F4	F5	F6		F7
	Ease-of-use	Tech. Quality	Bene-fits	Feed-back	HIE	Info quality	B2C collabo-ration	B2B Collabo-ration
The arrangement of fields and functions is logical on computer screen	0.698	0.203	0.121	0.094	0.077	0.141	0.055	0.121
Terminology on the screen is clear and understandable (for example titles and labels)	0.627	0.199	0.100	0.130	0.118	0.106	0.043	0.115
Entering and documenting patient data is quick, easy and smooth	0.574	0.350	0.187	0.162	0.171	0.080	0.021	0.120
The systems keep me clearly informed about what it is doing (for example saving data).	0.567	0.199	0.124	0.111	0.114	0.144	0.095	0.129
Routine tasks can be performed in a straight forward manner without the need for extra steps using the system	0.525	0.415	0.141	0.155	0.115	0.058	0.151	0.017
It is easy to obtain necessary patient information using the EHR system	0.519	0.282	0.212	0.084	0.216	0.194	0.061	0.118
Learning to use the electronic health record system does not require a lot of training.	0.458	0.272	0.085	0.078	0.113	0.035	0.007	-0.014
The information on the nursing record is in easily readable format	0.372	0.204	0.169	0.129	0.162	0.195	0.115	0.169
It is easy to correct mistakes (such as entry errors, ending up in the wrong screen, changing incorrect selections, etc.)	0.352	0.287	0.087	0.175	0.152	0.140	0.006	0.096
The reminders, alerts and warnings provided by the system are useful and are adequate.	0.318	0.128	0.263	0.186	0.134	0.193	0.161	0.067
The systems is stable in terms of technical functionality (does not crash, no downtime)	0.229	0.685	0.100	0.100	0.049	0.047	0.055	0.020
Faulty system function has caused or has nearly caused a serious adverse event for the patient.	-0.190	-0.595	-0.096	-0.151	-0.150	0.088	-0.072	-0.065
The system responds quickly to inputs	0.332	0.593	0.107	0.138	0.068	0.056	0.084	0.049
In my view, the system frequently behaves in unexpected or strange ways	-0.262	-0.584	-0.093	-0.089	-0.084	-0.085	0.008	-0.061
Information entered/documented occasionally disappears from the information system.	-0.106	-0.566	-0.046	-0.066	-0.078	-0.077	0.020	-0.115
Documentation of patient information for statistical purposes takes too much time.	-0.294	-0.347	-0.085	-0.140	-0.120	0.114	0.064	0.036
If I have problems with the system I can easily get help	0.212	0.342	0.141	0.267	0.215	0.074	0.023	0.190
Use of EHR systems frequently takes my attention away from the patient.	-0.250	-0.342	-0.236	-0.122	-0.175	0.023	0.023	-0.069
Information systems help to improve quality of care	0.201	0.210	0.708	0.106	0.082	0.106	0.018	0.161
Information systems help to ensure continuity of care	0.197	0.161	0.633	0.059	0.131	0.118	0.010	0.195
Information systems support compliance and adherence with the treatment recommendations	0.075	0.056	0.618	0.146	0.086	0.107	0.250	0.087
Information systems help in preventing errors and mistakes associated with medications	0.134	0.056	0.533	0.099	0.132	0.193	0.083	0.048
Information systems help to avoid duplicate tests and examinations	0.089	0.118	0.526	0.092	0.182	0.127	0.197	0.019
The EHR system provides me with information about the need for and effectiveness of treatment of my patients	0.092	0.007	0.224	0.129	0.036	0.162	0.639	0.067
The system supplier implements suggested corrections and amendments as wished	0.186	0.177	0.155	0.854	0.053	0.104	0.084	0.008
The system supplier is interested in feedback from users.	0.154	0.181	0.148	0.738	0.091	0.094	0.077	0.106
Suggestions for corrections and amendments are implemented sufficiently quickly	0.199	0.191	0.135	0.723	0.072	0.097	0.144	-0.016
I know to whom and how I can send feedback on the system, if I so wish	0.070	0.099	0.041	0.324	0.162	-0.033	0.034	0.189
Diagnostic imaging results are easily available on a regional level.	0.128	0.065	0.100	-0.023	0.467	0.335	-0.079	0.117

Laboratory results are easily available and are logically presented on a regional level	0.177	0.059	0.090	0.008	0.465	0.419	-0.017	0.051
Information on medications ordered in other organizations is easily available	0.156	0.146	0.175	0.122	0.437	-0.003	0.138	0.069
Obtaining patient information from another organization often takes too much time	-0.106	-0.188	-0.070	-0.108	-0.433	-0.006	-0.035	-0.015
Patient data (also from other organizations) are comprehensive, up-to-date and reliable	0.210	0.136	0.232	0.145	0.419	0.124	0.123	0.132
EHR systems support co-operation and communication between physicians working in different organizations	0.125	0.065	0.283	0.121	0.408	0.125	0.208	0.234
The patient's current medication list is presented in a clear format	0.103	0.014	0.216	0.053	0.080	0.568	0.005	0.085
The EHR system generates a summary view (e.g. on a timeline) that helps to develop an overall picture of the patients health status	0.178	0.022	0.104	0.077	0.050	0.510	0.199	0.020
The system monitors and notifies when the orders given to nurses have been completed	0.069	0.022	0.134	0.080	0.085	0.506	0.242	0.117
Measurement results provided electronically by the patient (e.g. via patient portal) help to improve the quality of care	0.027	0.014	0.084	0.054	0.063	0.108	0.594	0.000
EHR systems support co-operation and communication Between physicians and patients	0.187	0.095	0.250	0.153	0.162	0.018	0.316	0.295
EHR systems support co-operation and communication Between physicians and nurses	0.179	0.141	0.230	0.121	0.171	0.218	0.067	0.612
EHR systems support co-operation and communication Between physicians in your own organization	0.234	0.150	0.276	0.081	0.136	0.155	0.037	0.506

Extraction Method: Principal Axis Factoring.
Rotation Method: Varimax with Kaiser Normalization.
a. Rotation converged in 8 iterations.
